# Supplementary material for: Domestic and international mobility trends in the United Kingdom during the COVID-19 pandemic: an analysis of facebook data
Source: Int J Health Geogr. 2021 Dec 4;20:46. doi: 10.1186/s12942-021-00299-5 (PMC8643186; doi:10.1186/s12942-021-00299-5)

**Additional file 3**

**
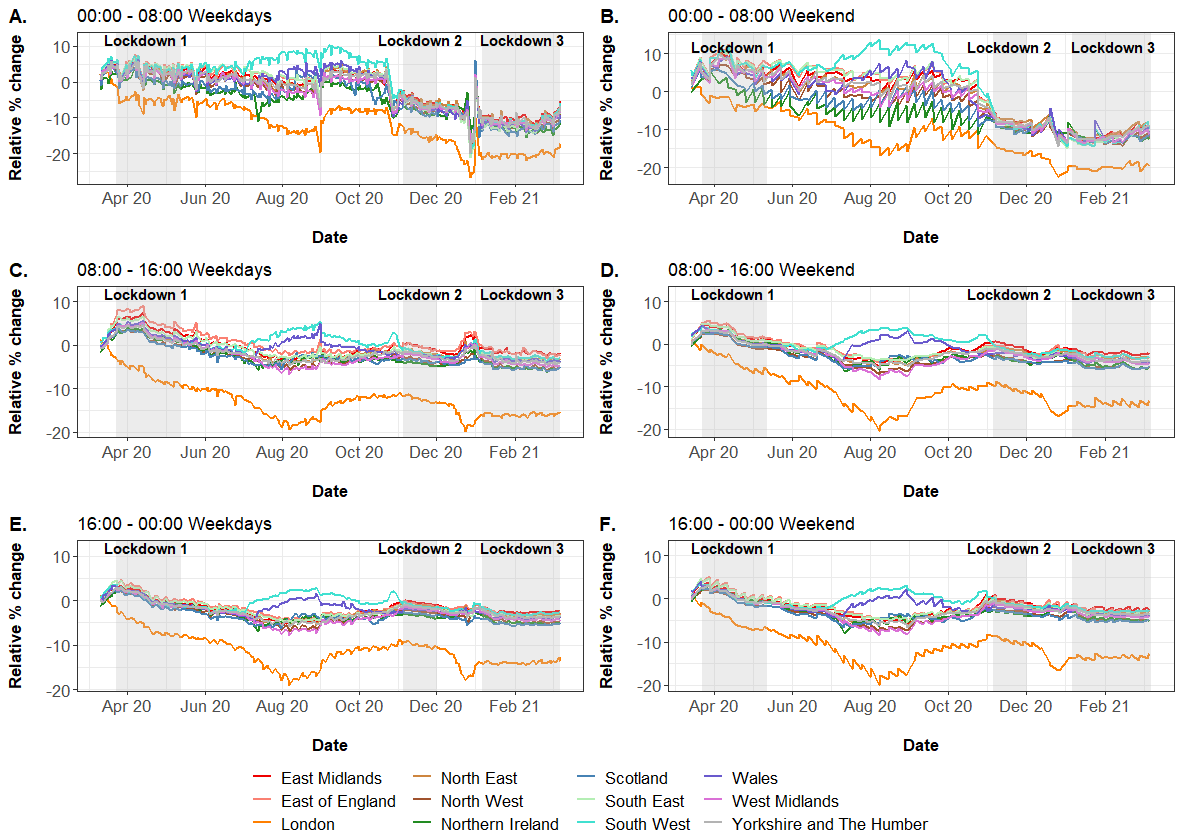
 Additional file 3: figure S1.** Percent change in population density of Facebook users within UK regions throughout 8-hour time periods on weekdays (A, C, E) and weekends (B, D, F) from March 10th 2020 and March 9th 2021. Regions are UK NUTS level 1 (Figure 1c). The change is relative to a pre-COVID baseline (see Methods for details). Shaded areas represent periods of national lockdown.

**
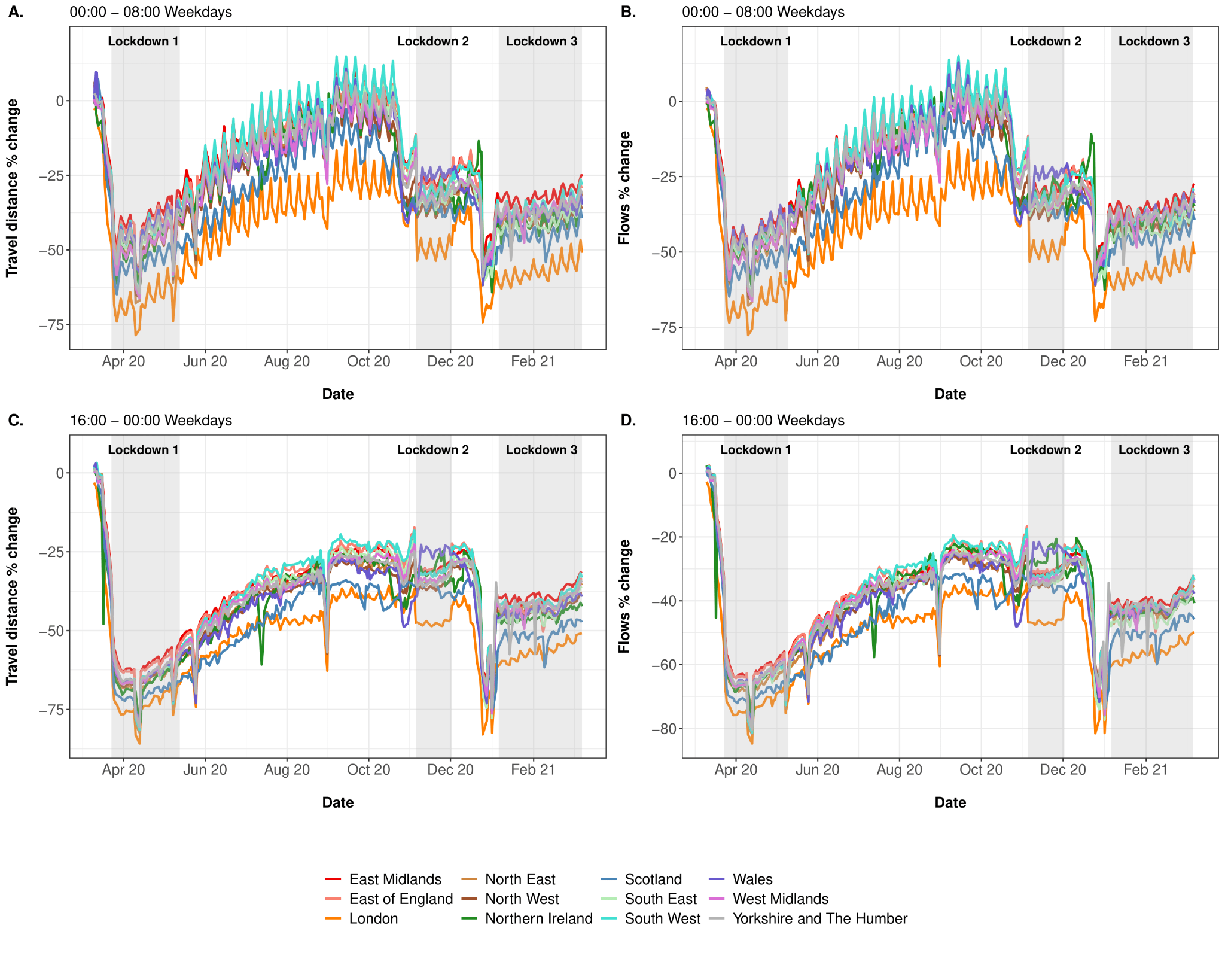
 Additional file 3: figure S2**. Percentage changes in (A, C) total distances travelled and (B, D) population movement (flows) for journeys between two adjacent 8-hour time periods on weekdays from March 10th 2020 to March 9th 2021. Regions are UK NUTS level 1 (Figure 1c). Time shown indicates the second 8-hour time period, where journeys ended. The change is relative to a pre-COVID baseline (see Methods for details). Shaded areas represent periods of national lockdown.

**
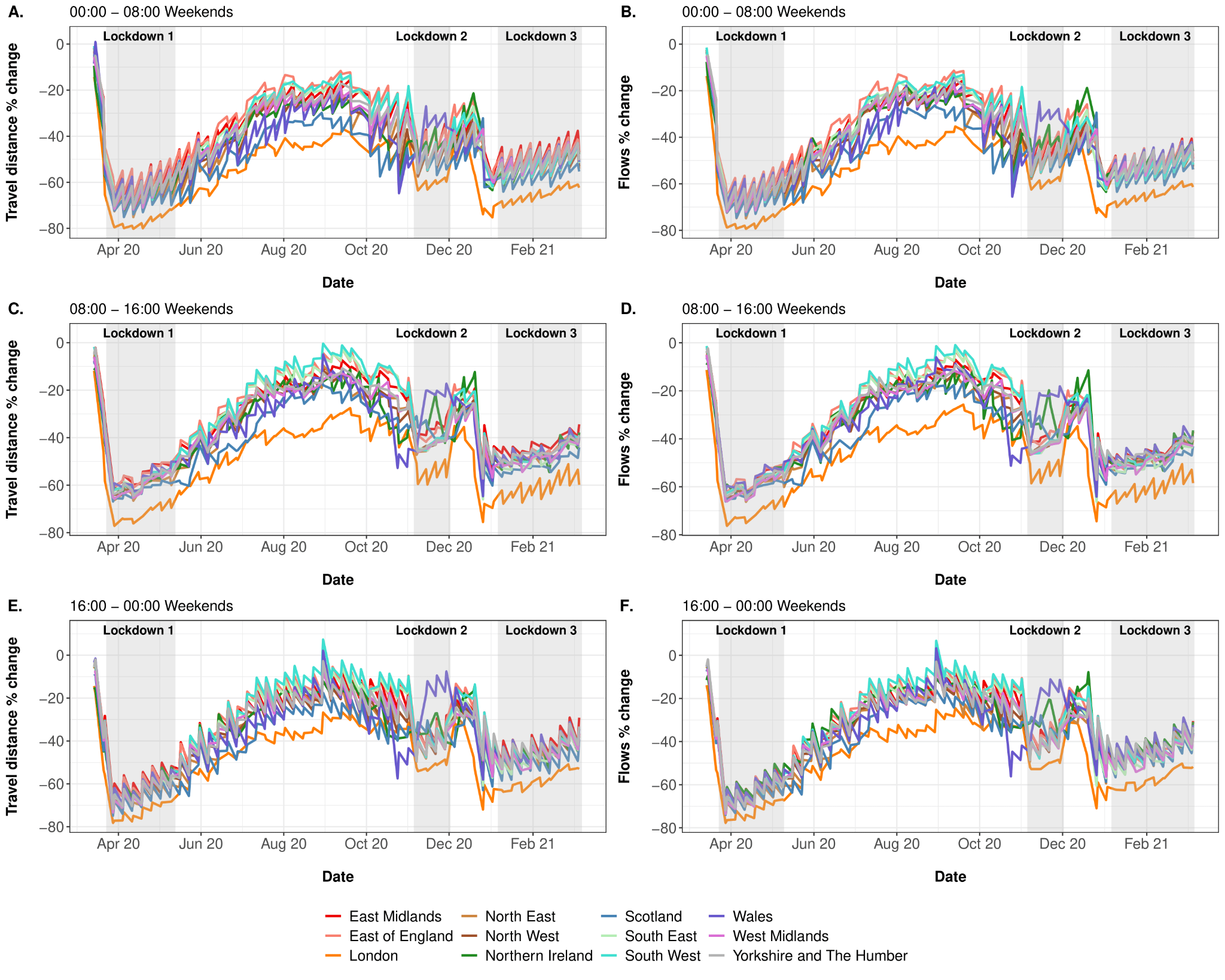
 Additional file 3: figure S3.** Percentage changes in (A, C, E) total distances travelled and (B, D, F) population movement (flows) for journeys between two adjacent 8-hour time periods on weekends from March 10th 2020 to March 9th 2021. Regions are UK NUTS level 1 (Figure 1c). Time shown indicates the second 8-hour time period, where journeys ended. The change is relative to a pre-COVID baseline (see Methods for details). Shaded areas represent periods of national lockdown.

**
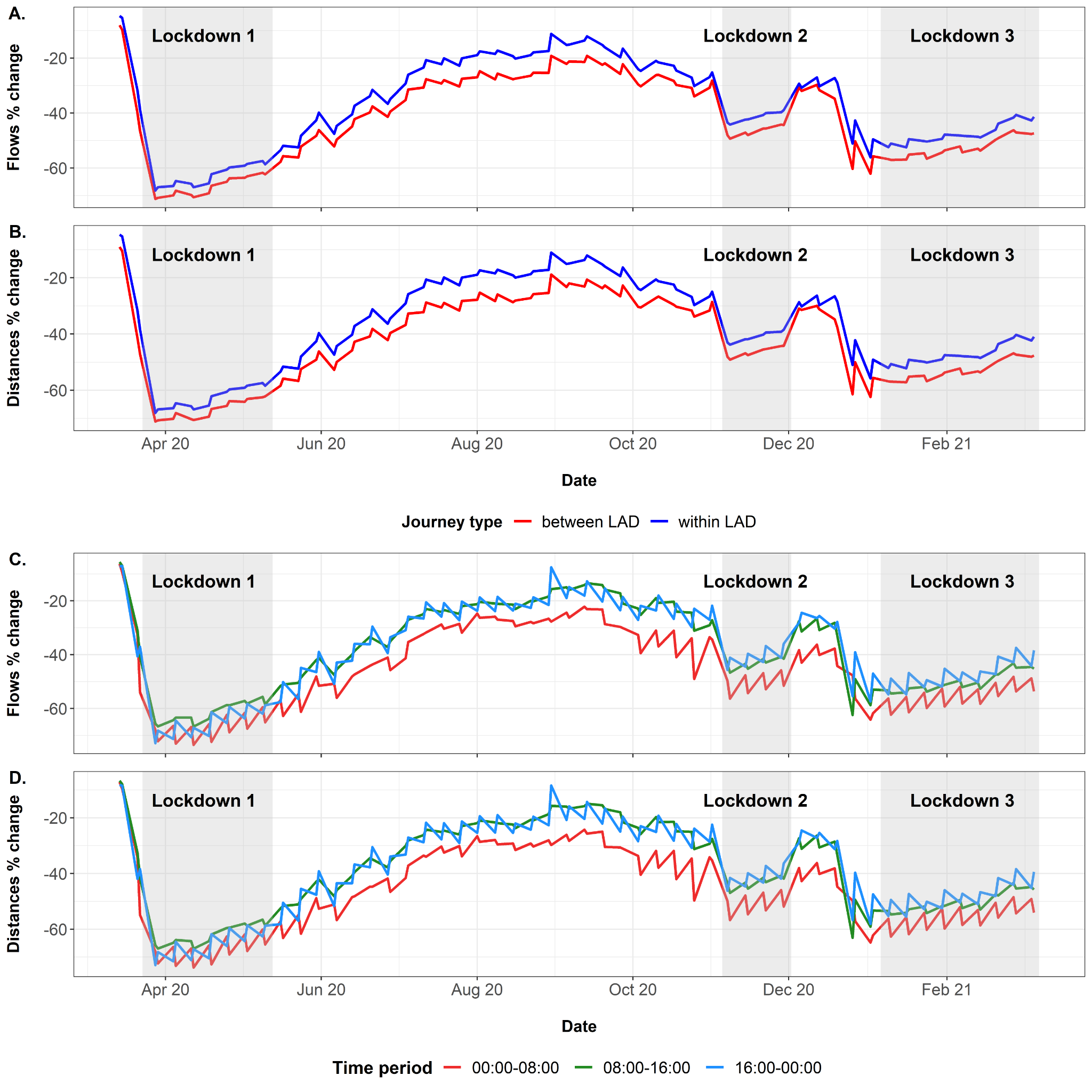
 Additional file 3: figure S4***.* Percentage changes in (A, C) daily movement (flows) of Facebook users and (B, D) total distance travelled from March 10th 2020 to March 9th 2021 on weekends. A and B show the changes corresponding to different journey types (between and within UK local authority districts). C and D show the changes corresponding to journeys ending within the different 8-hour time periods. The change is relative to a pre-COVID baseline (see Methods for details). Shaded areas represent periods of national lockdown.

**Additional file 3: table S1.** Summary statistics of weekly trend of mean log co-location probabilities for the NUTS level 1 regions in the UK from March 2020 to March 2021.

|  | **Min. (date)** | **Q1** | **Med.** | **Mean** | **Q3** | **Max. (date)** |
| --- | --- | --- | --- | --- | --- | --- |
| **East Midlands (England)** | -18.193 (14/04/2020) | -17.131 | -16.859 | -16.602 | -15.985 | -14.869 (10/03/2020) |
| **East of England** | -18.891 (14/04/2020) | -17.595 | -17.358 | -17.083 | -16.464 | -15.128 (10/03/2020) |
| **London** | -18.366 (14/04/2020) | -16.8 | -16.327 | -16.228 | -15.507 | -14.13 (03/03/2020) |
| **North East (England)** | -18.947 (14/04/2020) | -17.837 | -17.567 | -17.241 | -16.544 | -15.152 (10/03/2020) |
| **North West (England)** | -18.32 (14/04/2020) | -17.188 | -16.898 | -16.571 | -15.818 | -14.455 (10/03/2020) |
| **Northern Ireland** | -19.905 (31/03/2020) | -18.945 | -18.632 | -18.54 | -18.361 | -15.962 (03/03/2020) |
| **Scotland** | -19.076 (31/03/2020) | -18.23 | -17.945 | -17.702 | -16.948 | -15.423 (10/03/2020) |
| **South East (England)** | -18.691 (14/04/2020) | -17.584 | -17.304 | -17.023 | -16.396 | -14.996 (10/03/2020) |
| **South West (England)** | -18.718 (14/04/2020) | -17.618 | -17.25 | -17.019 | -16.256 | -15.044 (10/03/2020) |
| **Wales** | -18.446 (14/04/2020) | -17.696 | -17.391 | -17.151 | -16.589 | -15.219 (10/03/2020) |
| **West Midlands (England)** | -18.084 (14/04/2020) | -16.985 | -16.685 | -16.454 | -15.796 | -14.618 (10/03/2020) |
| **Yorkshire and The Humber** | -18.507 (14/04/2020) | -17.391 | -17.061 | -16.779 | -16.05 | -14.87 (10/03/2020) |

**Additional file 3: methods S1.** List of all Local Authority Districts that had no LAD allocated through the centroid method of assigning level 12 Bing tiles only.

Adur,

City of London,

Crawley,

Epsom and Ewell,

Gosport,

Hackney,

Islington,

Kensington and Chelsea,

Luton,

Oadby and Wingston,

Reading,

Richmond upon Thames,

Slough,

Southwark,

Tamworth,

Westminster.

**Additional file 3: methods S2**. Workflow for the ingestion, aggregation and analysis of Facebook population density Bing tile level 13 dataset (UK wide).


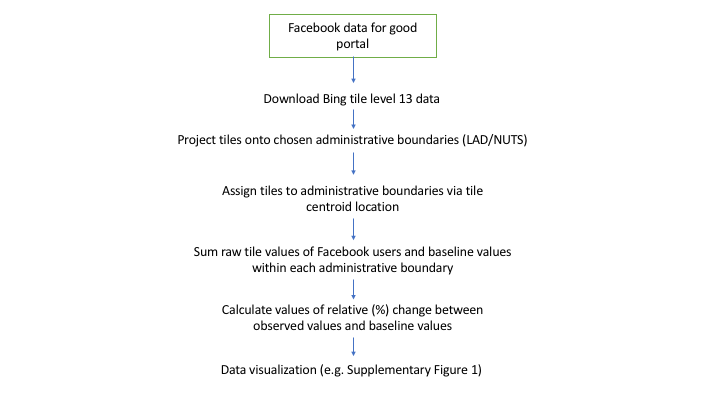


**Additional file 3: methods S3**. Workflow for the ingestion and analysis of Facebook population density Bing tile level 16 dataset (selected regions).

**
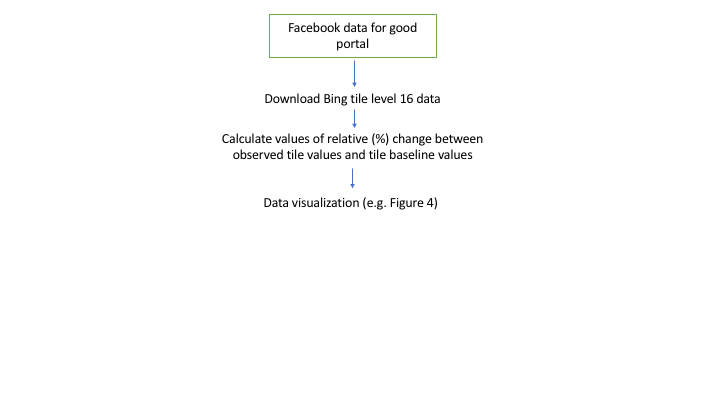
**

**Additional file 3: methods S4**. Workflow for the ingestion, aggregation and analysis of Facebook movement between tiles Bing tile level 12 dataset (UK wide).

**
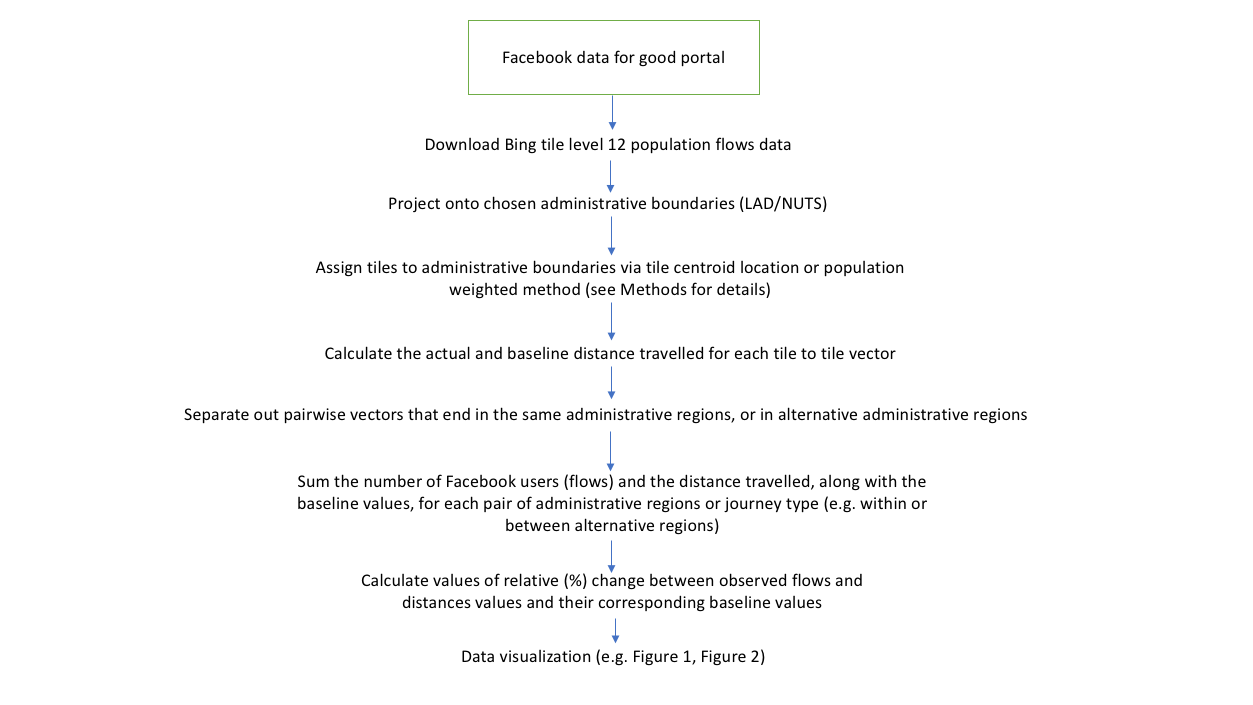
**

**Additional file 3: methods S5**. Workflow for the ingestion, aggregation and analysis of Facebook co-location dataset (UK wide).

**
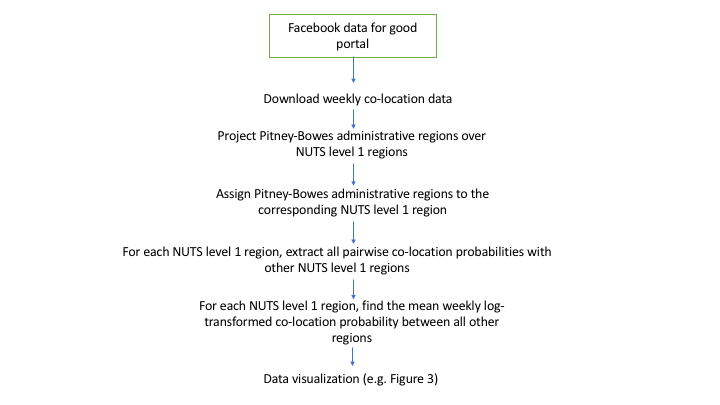
**

**Additional file 3: methods S6** Workflow for the ingestion, aggregation and analysis of Facebook international travel pattern dataset.


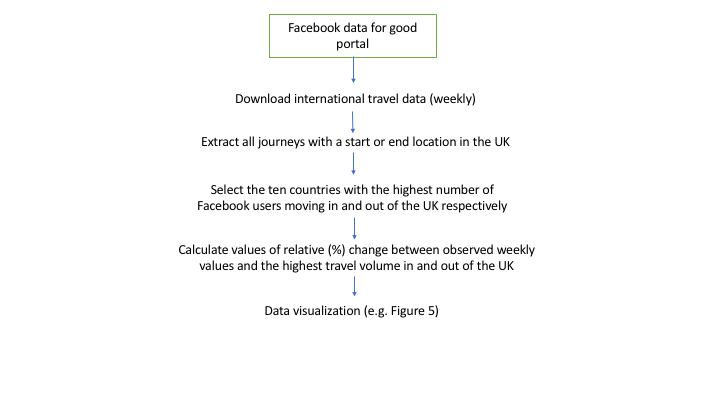

Supplement: Supplementary file 3 — Additional file 3. Supplementary methods, results and analysis of UK Facebook mobility data between 10th March 2020 and 9th March 2021; Workflow charts. [file 12942_2021_299_MOESM3_ESM.docx]
